# Stroke mortality attributable to high red meat intake in China and South Korea: An age–period–cohort and joinpoint analysis

**DOI:** 10.3389/fnut.2022.921592

**Published:** 2022-10-14

**Authors:** Fangyao Chen, Weiwei Hu, Shiyu Chen, Aima Si, Yuxiang Zhang, Jiaojiao Ma

**Affiliations:** ^1^Department of Epidemiology and Biostatistics, School of Public Health, Xi’an Jiaotong University Health Science Center, Xi’an, Shaanxi, China; ^2^Department of Radiology, First Affiliated Hospital of Xi’an Jiaotong University, Xi’an, Shaanxi, China; ^3^Department of Neurology, Xi’an Gaoxin Hospital, Xi’an, Shaanxi, China

**Keywords:** China, South Korea, age-period-cohort analysis, joinpoint analysis, high red meat intake, stroke mortality

## Abstract

The high intake of red meat is well recognized as a major health concern worldwide. It has been recognized as a risk factor for several non-communicable chronic diseases, including stroke. However, previously published studies have not performed a comprehensive analysis of the long-time trend of stroke mortality attributable to high red meat intake in China and South Korea, two countries with similar dietary patterns and changing trends. Therefore, this study aimed to reveal the influence of age, time period, and birth cohort on long-term trends of stroke mortality attributable to high red meat intake and relative gender differences in China and South Korea. Data were obtained from the Global Burden of Disease 2019 database. The age–period–cohort model was used to estimate the effect of age, time period, and birth cohort. The average and annual percent changes were estimated using the joinpoint regression analysis. Results indicated that the overall attributable age-standardized mortality rates of stroke in China decreased by 1.0% (*P* < 0.05) for female and 0.1% (*P* > 0.05) for male individuals, compared with a decrease of 4.9% for female and 3.7% for male individuals in South Korea (both *P* < 0.05). Age–period–cohort analysis revealed that the attributable stroke mortality decreased along with the time period, and increased along with age. Significant gender differences were observed, male individuals in both countries were at higher risk than their female counterparts, especially in China. Joinpoint analysis suggested that the attributable stroke mortality for both genders in South Korea and female individuals in China showed a decreasing trend, while it is stable for male individuals in China. Although prominent reductions were observed during the past decades, the attributable stroke mortality risk in China and South Korea is still high. Our findings indicate that controlling the intake of red meat may be a cost-effective strategy to reduce stroke mortality risk and the corresponding disease burden, especially for Chinese male individuals.

## Introduction

Stroke is the second leading cause of death and the third leading cause of disability worldwide (1). Compared to 1990, the number of patients with stroke increased by 70%, and stroke-related death increased by 43%, reaching 12.2 million and 6.55 million in 2019, respectively. Although the global age-standardized incidence rate (ASIR) and prevalence rate (PR) of stroke decreased, the ASIR and PR of stroke for people younger than 70 years increased by 15 and 20%, respectively (2). From a regional perspective, East Asia had the highest ASIR for ischemic stroke in 2019 and the largest increase in the ASIR from 1990 to 2019 (3). With high mortality and morbidity rates, stroke is a serious threat to human survival and quality of life. Therefore, it is imperative to develop strategies to prevent and reduce the risk of stroke.

The incidence of cardiovascular and cerebrovascular diseases is usually closely related to an unhealthy diet structure, unreasonable living habits, and other diseases (4). Among them, people who like greasy, fried, and high-sugar foods are at high risk of such diseases (5). Globally, China is second after Egypt with pronounced increases in the ASIR of stroke, and in South Korea, stroke has long been the second leading cause of death after cancer (6). In the past three decades, over 11 million people died due to dietary risk factors of non-communicable diseases (7). Published studies have suggested that dietary factors have a positive association with the increase in stroke risk (8). Consuming diets high in salt (9) and lacking vegetables or fruit (10), drinking alcohol (11), low whole grain intake (12), and high red or processed meat intake (13) were all found to be positively associated with high stroke risk. A recent study found that high consumption of red meat might result in an increased risk of stroke (14), and a previous study also revealed that high consumption of red meat was associated with all-cause mortality (15). A previous study has confirmed that an unbalanced diet pattern, including high in red meat, was a risk factor for stroke-related death (16).

Both China and South Korea are countries with high standardized stroke and DALYs in the East Asia region (17). Stroke mortality risk was well acknowledged to be associated with diet factors (18). China and South Korea share a similar traditional dietary pattern. The diets of China and South Korea mainly comprise grains traditionally, with rice being the main food in the south, and noodles being the main food in the north (19, 20). Although several studies have indicated that the traditional dietary patterns in China and South Korea may be associated with low stroke mortality risks (21, 22), dietary patterns in both countries have changed in recent years. During the past several decades, with the development of socioeconomic status and the influence of Western dietary patterns, the diets in both the countries have gradual shifted to a pattern based on meat and milk (23–25), and the intake of meat, especially the consumption of red meat, has increased significantly (26).

The same tradition in dietary patterns and similar changing trends make China and South Korea have good comparability in analyzing stroke mortality attributable to dietary factors. Through literature review, we found that studies on the long-time trend of dietary factors attributable to stroke mortality have mostly focused on the intake of salt, vegetable, fruit, and alcohol (27); therefore, this study aimed at the long-time trend of high red meat intake (HRMI) to attributable stroke mortality.

Therefore, in this study, we aim to analyze the long trend of high red meat intake attributable to stroke mortality in China and South Korea—two Asian countries with similar eating habits, traditions, and changing trends—and the corresponding gender difference during the period from 1990 to 2019. Research data were obtained from the Global Burden of Disease (GBD) 2019 study, and we used the age–period–cohort (APC) frame to explore the effects of age, year period, and birth cohort, as well as the long time trends.

## Materials and methods

### Data source

Research data were obtained from the GBD 2019 study using the Global Health Data Exchange Tool.^1^ In the GBD study, all data were expressed as numerical values and their 95% confidence intervals. The GBD 2019 study is a comprehensive health loss study using a harmonized and comparable approach to the burden of disease by gender and age-group in 195 countries and territories based on reliable and representative data from multiple global sources from 1990 to 2019 (28).

### Measurements definition

In the GBD 2019 study, diets high in red meat are defined as an average daily consumption (in grams) of red meat (including beef, pork, lamb, and goat, but excluding poultry, fish, eggs, and all processed meats) higher than 23 (18–27) grams (28).

Stroke mortality was defined according to the WHO International Statistical Classification of Diseases and Related Health Problems, Ninth (ICD-9) (29) or Tenth Revision (ICD-10) (30). In the GBD 2019 study, the data on stroke mortality in China were collected from the Disease Surveillance Points, the Maternal and Child Surveillance System, the Chinese Center for Disease Control and Prevention Cause of Death Reporting in China (31). The stroke mortality rates in the Republic of Korea were collected through the civil registration and vital statistics, registration of death in the Population Register System, and the National Bureau of Statistics (32). The manner in which the relevant data were collected and the sources of the data in the GBD study ensured the reliability of the data (27, 33), and the reliability of its measurement has also been discussed and acknowledged in other studies (27, 33, 34).

The attributable mortality was estimated using the population-attributable fraction (PAF) considering genders, each year, and age-groups in GBD 2019 study. The PAF was the proportion of stroke mortality reduced if HRMI was reduced to the level of theoretical minimum risk exposure level (TMREL). For stroke mortality attributable to HRMI, the TMREL refers to the average daily consumption of red meat of no more than 23 grams (31). In the GBD 2019 study, the PAF was defined as follows (32):


$${\text{PAF}}_{jcasgt}=\frac{\int_{x=l}^{u}RR_{jcasp}\;(x)\;P_{jaspt}\;(x)\;dx\;-\;RR_{jcasp}(TMREL_{jas})}{\int_{x=l}^{u}RR_{jcasl}\;(x)\;P_{jaspt}\;(x)\;dx}$$

Here, PAF_*joasgt*_ is the PAF for cause (of death) *c*, for age-group *a*, sex *s*, location *p*, and time period (usually year) *t*; *RR*_*jcasl*_(*x*) is the relative risk of exposure level *x* for risk factor *j*, for cause *c*, controlling age *a*, sex *s*, and location *p*; *l* and *u* were the lowest and highest observed exposure levels (the lower and the upper boundary) of *x*; and *TMREL*_*jas*_ is the TMREL for risk factor *j*, age-group *a*, and sex *s*. For the HRMI attributable stroke mortality, the risk factor is HRMI, the cause is stroke, and the location is China and South Korea, respectively. In the GBD 2019 study, stroke mortality attributable to HRMI was defined as the (basic) stroke mortality multiplied by the PAF (32).

The age-standardized mortality rates (ASMRs, per 100,000 population) were also analyzed in this study. In the GBD 2019 study, it was defined as follows:


$$\text{ASMR}=\left(\frac{\sum a_{i}\,w_{i}}{\sum w_{i}}\right)\times 100,000$$

Here, *a*_*i*_ was the specific age ratio. The weights *w*_*i*_ were derived from the GBD 2019 global standard population (32).

### Statistical analysis

Graphs were generated with R programming language (version 4.0.3, The R Foundation) and RStudio (Version 1.1.463, RStudio Inc., MA, USA) software. All statistical tests were two-tailed, and the level of significance was set at 0.05.

To estimate the effect of age, year period, and birth cohort, we performed the age-period-cohort (APC) model using the APC Web Tool^2^ (35). To convert the data to fulfill the requirement of the APC model, age-specific stroke mortality rates attributable to HRMI were organized into consecutive 5-year periods from 1994 to 2019 and successive 5-year age-groups from the age of 25 to 29 years to 90 to 94 years. Since the data of population groups under 25 years were not available in the GBD 2019 database and population higher than 94 years was recorded as one group (thus cannot convert to 5-year age-groups), those age-groups were not included in the analysis.

We estimated coefficients for age, period, and cohort effects for attributable stroke mortality using the APC model. The APC model can be expressed as follows (35, 36):


R=c+α⁢A⁢g⁢e+β⁢P⁢e⁢r⁢i⁢o⁢d+γ⁢C⁢o⁢h⁢o⁢r⁢t+ε

Here, *R* represents the mortality rate, *ε* is the residual, and *c* is the constant; *Age*, *Period*, and *Cohort* represent the three factors including age-group, year period, and birth cohort, which may affect the mortality; and *α*, *β*, and *γ* are the coefficients of the three factors, representing the effect of age-group, year group, and birth cohort. In the APC analysis, the exponential values of the coefficients denote the relative risk (RR) of attributable stroke morbidity of a given age, period, and birth cohort, relative to the reference groups (36, 37). The 95% CI of RR was also estimated in APC analysis.

We also used the APC model to estimate the net and local drifts, as well as their corresponding 95% CI, which show the overall log-linear trend by year period and birth cohort. The local drift represents the changes in the attributable mortality rate from the previous year to the current year, while the net drift represents the overall changes in the attributable mortality rate from 1994 to 2019 (35, 36). A local drift estimation > 0 means the attributable mortality rate increased from the previous year to the current year.

Considering the nonlinear trend of the ASMR and crude mortality rate (CMR) across time periods, in this study, we performed the joinpoint regression analysis to estimate the long-time trends of stroke mortality attributable to HRMI. The joinpoint regression was also called change-point regression, which was first introduced by Kim et al. (38). It can be defined as follows:


$$\log\left(y|x\right)=c+\beta \,Y\,e\,a\,r+\delta_{1}\,{\left(x-\tau_{1}\right)}^{+}+\cdots +\delta_{k}\,{\left(x-\tau_{k}\right)}^{+}$$

Here, *y* represents the mortality rate, *Year* is the observation year, β represents the coefficient of *Year*, τ_*k*_ is the unknown turning time point (joint point) that needs to be identified, and *c* is the constant.

To determine the magnitude of trends in attributable stroke mortality, the annual percent change (ANPC), average annual percent change (AAPC), and its corresponding 95% confidence interval (CI) were estimated by joinpoint regression analysis. The ANPC and AAPC were defined as follows:


$${\text{ANPC}}_{i}=(e^{b_{i}}-1)\times 100\%$$

$$\text{APPC}=\left(e^{\frac{\sum b_{i}w_{i}}{\sum w_{i}}}-1\right)\times 100\%$$

Here, *b_i_* is the estimation of β on the *i*th identified trend and *w_i_* is the length of the *i*th identified trend.

The joinpoint model analysis was conducted by the Joinpoint Regression Program (version 4.9.0.0, March 2021; Statistical Research and Application Branch, NCI, USA).

## Results

### Long-time trend of attributable stroke age-standardized mortality rate

**FIGURE 1:**
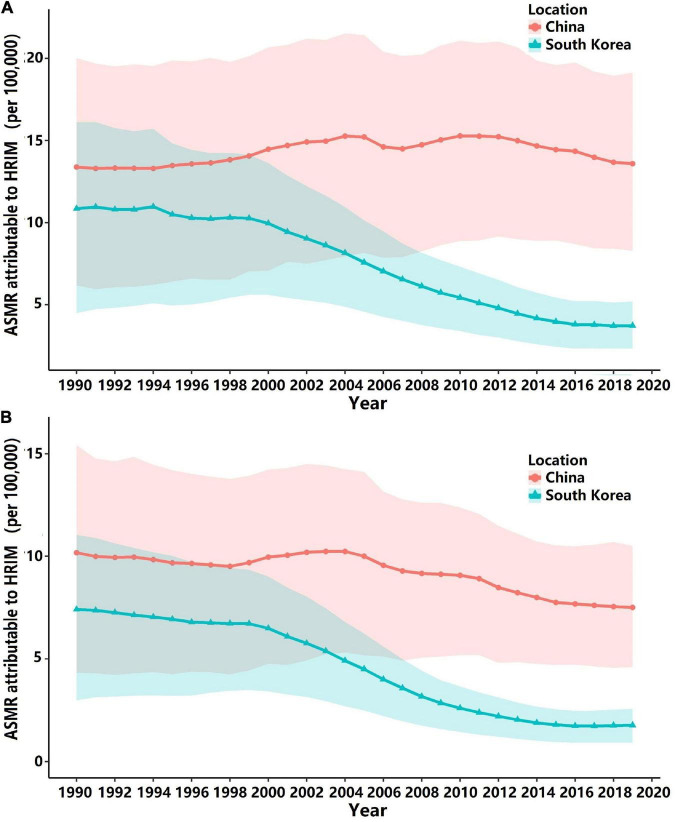
Age-standardized mortality rates (ASMRs) of stroke attributable to HRMI (per 100,000) and corresponding 95% confidence intervals (presented with shadows) for both genders in China and South Korea. **(A)** For male individuals in China and South Korea; **(B)** for female individuals in China and South Korea.

The long-term trend of stroke ASMRs attributable to HRMI by gender in China and South Korea is presented in Figure 1. As shown in Figures 1A,B, the attributable ASMRs for both genders showed an overall increasing trend from 1990 to 2019. To be specific, the ASMR for male individuals in South Korea (Figure 1A) decreased from 10.847 (95% CI: 4.493∼16.409) to 3.715 (95% CI: 2.251∼5.202) per 100,000, and that for female individuals decreased from 7.408 (95% CI: 2.891∼11.041) to 1.768 (95% CI: 0.909∼2.564) per 100,000 (Figure 1B).

The stroke ASMR attributable to HRMI in China is higher than that in South Korea for both genders (all *P*-values > 0.05 after the year 2008), and the attributable ASMRs in China for male individuals showed an overall relatively stable trend (*P* > 0.05) from 13.383 (95% CI: 6.166∼20.008) to 13.587 (95% CI: 8.278∼19.130), while for female individuals, ASMRs slightly decreased (thought *P* > 0.05) from 10.179 (95% CI:4.328∼15.416) to 7.513 (95% CI: 4.603∼10.498) per 100,000, as shown in Figure 1.

### Local and net drifts

**FIGURE 2:**
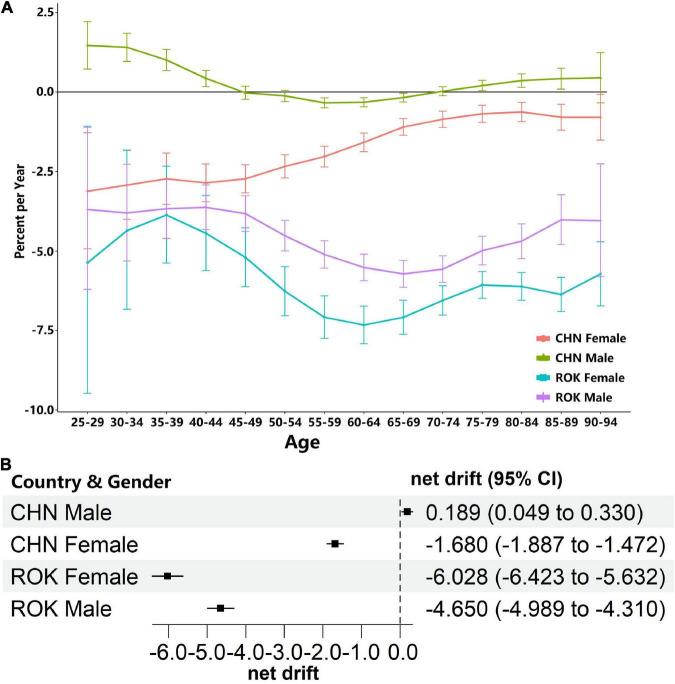
Local and net drifts of HRMI attributable stroke mortality (error bars stand for 95% confidence intervals) for both genders in China (CHN) and South Korea (SK) from 1994 to 2019. **(A)** The local drifts for both genders in China and Korea along with age; **(B)** net drifts for both genders in China and Korea from 1994 to 2019.

The local and net drifts of attributable stroke mortality in China and South Korea are shown in Figure 2. As shown in Figure 2A, the local drifts for male and female individuals in China show an upward trend along with age-groups (though all < 0). The peak of the local drift for Chinese male individuals (1.468, 95% CI: 0.728∼2.214) appears in the age-group of 25–29 years, while that for Chinese female individuals and both genders in South Korea showed a relatively stable trend (all below 0), as shown in Figure 2A.

As shown in Figure 2B, the net drift is 0.189% (95% CI: 0.048∼0.330%) for Chinese male individuals, −1.680% (95% CI: −1.887∼−1.472) for Chinese female individuals, −4.650 % (95% CI: −4.989∼−4.310%) for South Korea male individuals, and −6.028 % (95% CI: −6.424∼−5.632%) for female individuals in South Korea.

### Age–period–cohort analysis

**FIGURE 3:**
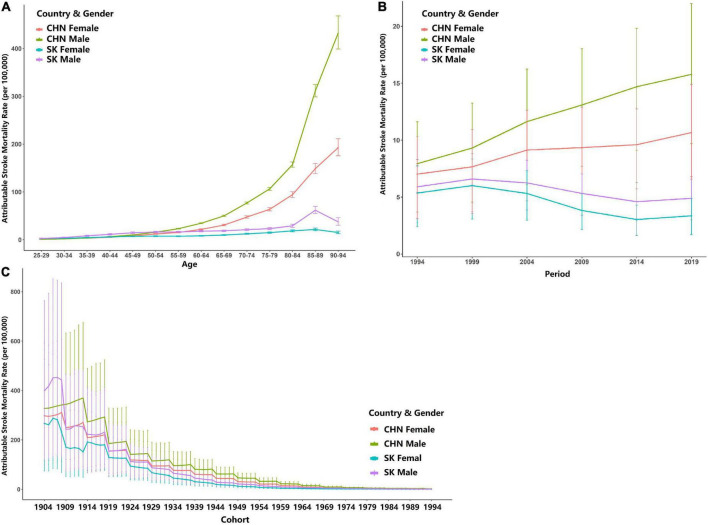
Longitudinal curve for age, time period, and birth cohort for both genders in China (CHN) and South Korea (SK). **(A)** Longitudinal age curves; **(B)** longitudinal period curve; **(C)** longitudinal cohort curve (error bars stand for 95% confidence intervals).

Figure 3A shows the longitudinal age curve (adjusted for period and cohort effects) of HRMI attributable stroke mortality rates by gender and country. In general, the attributable stroke mortality rates (per 100,000) increased for both genders in China and South Korea. As shown in Figure 3A, for both genders in China, the attributable stroke mortality rates increased rapidly after the age-group of 55–59 years. The attributable mortality rates for male individuals in China reached a maximum level of 432.104 (95% CI: 398.834∼468.150) per 100,000 at the age of 90–94 years, while for female individuals in China, the mortality rates increased to 192.633 (95% CI: 175.584∼111.339) per 100,000 in the age-group 90–94 years. The longitudinal age curve showed no sign of decrease for both genders in China. For both genders in South Korea, the attributable stroke mortality rates reached their peaks at the age-group 85–89 years by 61.709 (95% CI: 54.653∼69.677) per 100,000 for male individuals and 21.446 (95% CI: 18.852∼24.397) per 100,000 for female individuals and then both decreased slightly.

Figure 3B shows the longitudinal curve of the period (adjusted for age and cohort effect). A continuous upward trend in the stroke mortality rate attributable to HRMI can be observed for both genders in China, while that for both genders in South Korea was stable and showed a slightly upward and then downward trend. The peaks for the attributable stroke mortality rates for both genders in South Korea appeared in the year period of 1999, with 6.005 (95% CI: 3.063∼8.344) per 100,000 for female individuals and 6.584 (%%CI: 0.716∼8.807) per 100,000 for male individuals.

Figure 3C shows the longitudinal curve of the birth cohort (adjusted for age and year period). A continuous downward trend can be observed for both genders in China and South Korea.

**FIGURE 4:**
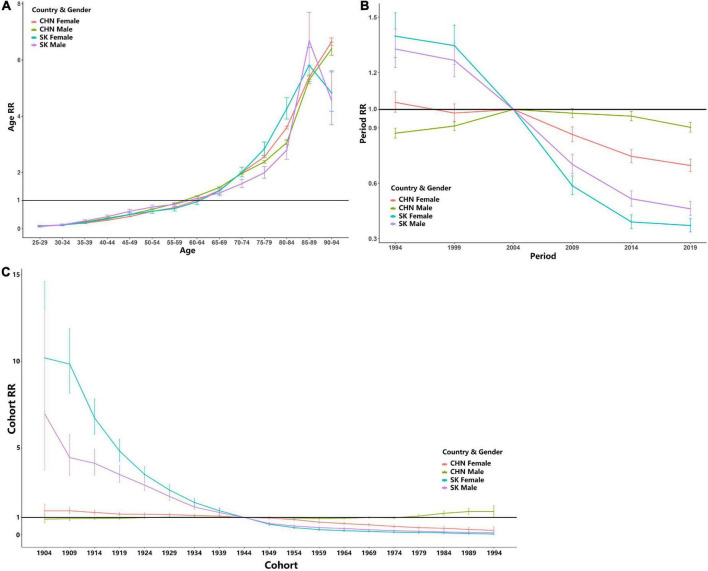
Long-term effects of age, time period, and birth cohort for both genders in China (CHN) and South Korea (SK). The reference line (RR = 1) was also plotted. **(A)** Age RR; **(B)** period RR; **(C)** cohort RR (error bars stand for 95% confidence intervals).

The effect of age by gender and country is shown in Figure 4A. In general, we could observed an overall upward trend for both genders in China and South Korea. For both genders in China, the peaks of age RR appeared at the age-group of 90–94 years with 6.395 (95% CI: 6.166∼6.633) for male individuals and 6.646 (95% CI: 6.614∼6.781) for female individuals. For both genders, in South Korea, the peak appeared at the age-group of 85–89 years, with 6.677 (95% CI: 5.7994∼7.693) for male individuals and 5.808 (95% CI: 4.239∼6.439) for female individuals and then decreased slightly for both genders.

The effect of the time period by gender and country is shown in Figure 4B. The period RR for both genders in China shows an relative stable trend. The period RR for female Chinese in 1994 was above 0 (1.0384, 95% CI: 0.985∼1.095), while no statistical significance was observed. An upward and then downward trend was observed for the period RR for Chinese male individual, while the estimated RR for Chinese male individuals was no higher than 1. While for South Korea, the estimated period RR for female individuals showed an overall downward trend, and the decline became more rapid after the year 1999. The period RR for both genders in South Korea showed an inverse “S” shape, and a continuous decreasing trend was observed.

The estimated cohort RRs by gender and country are shown in Figure 4C. The cohort RR for both genders in China was relatively stable, while that for South Korea showed a downward trend along with the birth cohort. The cohort RR for female individuals in South Korea dropped from 10.187 (95% CI: 7.106∼14.604) in the birth cohort in 1904 to 0.046 (95% CI: 0.108∼0.196) in the birth cohort in 1994, while that for male individuals in South Korea was from 6.964 (95% CI: 3.732∼12.991) in 1904 to 0.115 (95% CI: 0.049∼0.270) in 1994.

### Joinpoint analysis

The nonlinear long-time trends of stroke mortality attributable to HRMI by gender and country were fitted through the joinpoint regression analysis. Detailed results are presented in Table 1.

**TABLE 1:** Annual percent change of crude and age-standardized mortality attributable to high red meat intake by gender and country from 1990 to 2019.

Country	Segment		Crude mortality rates			Age-standardized mortality rates		
				
			Year	ANPC (95% CI)	*t* (*P*)	Year	ANPC (95% CI)	*t* (*P*)
China	Female	Trend 1	1990∼1997	0.8(0.5, 1.1)	5.3(<0.001)	1990∼1997	−0.9(−1.1, −0.6)	−6.9(<0.001)
		Trend 2	1997∼2004	3.6(3.3, 3.9)	24.1(<0.001)	1997∼2004	1.1(0.8, 1.5)	7.7(<0.001)
		Trend 3	2004∼2007	−0.9(−2.5, 0.7)	−1.2(0.257)	2004∼2007	−3.7(−5.4, −1.9)	−4.4(0.001)
		Trend 4	2007∼2010	2.2(0.7, 3.8)	3.1(0.009)	2007∼2010	−0.5(−2.4, 1.4)	−0.6(0.545)
		Trend 5	2010∼2015	0.1(−0.4,0.5)	0.3(0.767)	2010∼2015	−3.2(−3.8, −2.6)	−11.1(<0.001)
		Trend 6	2015∼2019	2.6(2.2,3.1)	12.5(<0.001)	2015∼2019	−0.8(−1.5, 0)	−2.3(0.04)
		AAPC	–	1.6(1.3, 1.8)	12.3(<0.001)	–	−1.0(−1.3, −0.7)	−6.9(<0.05)
	Male	Trend 1	1990∼1996	1.7(1.2, 2.1)	8.2(<0.001)	1990∼1996	0.2(−0.1, 0.6)	1.4(0.191)
		Trend 2	1996∼2004	4.3(4.1, 4.6)	36.4(<0.001)	1996∼2004	1.6(1.4, 1.9)	14.9(<0.001)
		Trend 3	2004∼2007	1(−0.4, 2.5)	1.5(0.155)	2004∼2007	−1.8(−3.4, −0.3)	−2.5(0.026)
		Trend 4	2007∼2011	4.2(3.5, 4.8)	13.6(<0.001)	2007∼2011	1.6(0.8, 2.3)	4.3(0.001)
		Trend 5	2011∼2019	1.3(1.2, 1.5)	22.9(<0.001)	2011∼2019	−1.6(−1.8, −1.4)	−19.5(<0.001)
		AAPC	−	2.6(2.4, 2.8)	25.8(<0.001)	-	0.1(−0.1, 0.3)	0.1(>0.05)
South Korea	Female	Trend 1	1990∼1999	2.3(2.2, 2.4)	46.9(<0.001)	1990∼2000	−1.3(−1.4, −1.1)	−20.6(<0.001)
		Trend 2	1999∼2002	−1.1(−2, −0.2)	−2.6(0.024)	2000∼2004	−6.5(−7.4, −5.6)	−14.9(<0.001)
		Trend 3	2002∼2005	−4.3(−5.3, −3.4)	−9.5(<0.001)	2004∼2010	−10.5(−11.1, −9.9)	−34(<0.001)
		Trend 4	2005∼2009	−7.0(−7.6, −6.4)	−24.8(<0.001)	2010∼2015	−7.3(−8.9, −5.7)	−9.5(<0.001)
		Trend 5	2009∼2015	−4.4(−4.8, −4)	−24.9(<0.001)	2015∼2019	0(−2.1, 2.1)	0(0.98)
		Trend 6	2015∼2019	3.5(2.8, 4.1)	12.1(<0.001)			
		AAPC	–	−1.3(−1.5, −1.1)	−14.0(<0.001)	–	−4.9(−5.3, −4.4)	−22.9(<0.001)
	Male	Trend 1	1990∼1999	2.7(2.5, 2.9)	25.4(<0.001)	1990∼1999	−0.8(−1.1, −0.5)	−5.8(<0.001)
		Trend 2	1999∼2003	−1.0(−1.9, −0.1)	−2.2(0.038)	1999∼2003	−4.1(−5.6, −2.6)	−5.6(<0.001)
		Trend 3	2003∼2016	−2.9(−3, −2.7)	−42.8(<0.001)	2003∼2015	−6.5(−6.8, −6.1)	−40.5(<0.001)
		Trend 4	2016∼2019	4.0(2.5, 5.5)	5.8(<0.001)	2015∼2019	−1.3(−3.8, 1.3)	−1(0.311)
		AAPC	–	−0.2(−0.4, 0.0)	2.0(<0.1)	–	−3.7(−4.1, −3.3)	−17.5(<0.001)

As for the CMRs of female individuals in China, there are five trends, as shown in Table 1. The average increase (AAPC) is 1.6% (95% CI: 1.3%∼1.8%) per year from trend 1 to trend 5 (1990∼2019), indicating an overall upward change. The peak of growth appears in trend 2 from 1997 to 2004 at 3.6% (95% CI: 3.3%∼3.9%) per year. For the ASMRs of female individuals in China, the overall trends are downward from 1990 to 2019. The AAPC is −1.0 (95% CI: −1.3∼−0.7). Except for trend 2 (by 1.1% per year), the ASMRs show a consistent downward trend.

For the CMRs of male individuals in China, the overall trend is upward and the AAPC is 2.6% (95% CI: 2.4%∼2.8%) per year, as shown in Table 1. The peak appears in trend 2 from 1996 to 2004 by 4.3% (95% CI: 4.1%∼4.6%), and the upward trend is consistent across all time periods. For the ASMRs of male individuals in China, the overall trend is relatively stable with an AAPC equal to 0.1 (95% CI: −0.1∼0.3).

The CMRs of male individuals in South Korea show an inconsistent trend from 1990 to 2019. The overall AAPC is −0.2% (95% CI: −0.4%∼0.0%) per year, as shown in Table 1. During the first time period (1990∼1999), the CMRs increased by 2.7% (95% CI: 2.5%∼2.9%), and then it decreased by −1.0 (−1.9%∼−0.1%) per year during 1999∼2003 and −2.9% (95% CI: −3%∼−2.7%) per year during 2003∼2016. However, it increased again by 4.0% (95% CI: 2.5%∼5.5%) per year during 2016∼2019. The ASMR shows a significant and consistent downward trend of −3.7% (95% CI: −4.1%∼−3.3%) per year. The lowest point appears in 2003∼2005 at −6.5% (95% CI: −6.8%∼−6.1%).

Overall, for both CMRs and ASMRs, there are downward trends for both genders in South Korea and an upward trend for male individuals in China. Detailed results are presented in Table 1.

## Discussion

Stroke has long been identified as one of the leading cause of death worldwide (39). It was well acknowledged that diet factors were associated with the risk of stroke mortality (18). In the current study, we have analyzed the long-time trend of HRMI attributable stroke mortality in China and South Korea using APC analysis and joinpoint regression. We also found this study is the first research focusing on the long-time trends of stroke mortality attributable to HRMI and providing a comprehensive quantified comparison between China and South Korea.

The result of our study was consistent with previously proposed idea that the increase in red meat intake is associated with increased risk of stroke (40, 41). Although the relationship between red meat intake and stroke has not been fully studied yet, there are several possible underlying mechanisms about how red meat may affect the initiation and development of stroke. First, red meat is a major source of carnitine, which is metabolized to trimethylamine (TMA) *via* gut flora and then further oxidized to trimethylamine N-oxide (TMAO) by flavin monooxygenase (FMOS) (42). Several studies have proved that higher serum TMAO was associated with increased risk of first stroke and the severity of stroke (43, 44). Previous studies also demonstrated that HRMI is associated with stroke or cardiovascular disease risk, presumably owing to increased foam cell formation, decreased reverse cholesterol transport, and enhanced platelet aggregation (45, 42).

Second, iron is also abundant in red meat (46). Most of the iron in red meat exists in the form of heme iron (47). Excess heme iron intake in red meat may result in metabolic syndrome (MetS) *via* the oxidative injury mechanism (48), which may increase the risk of stroke (49). Moreover, a prospective cohort study conducted with Sweden men indicated that high heme iron intake was positively associated with stroke risk (50). Thus, carnitine and heme iron in red meat may contribute to the link between high red meat intake and stroke risk.

In the APC analysis, we found that the local drift of both genders in South Korea was below 0 in all age-groups, indicating a stable decreasing trend for the stroke mortality rate attributable to HRMI. While for male individuals in China, the local drift is higher than 0 before the age-group of 45–49 years and after the age-group of 65–69 years. The local drifts for female individuals in China was also below 0 for all age-groups. The overall net drifts for both genders in South Korea and female individuals in China suggested a downward trend, while that for male individuals in China is above 0. The local and net drifts indicated that for male individuals in China, especially the middle-aged Chinese male individuals, the stroke risk is upraising.

This study also observed that the longitudinal age curves of stroke mortality attributable to HRMI were growing faster for Chinese people than for Korean people with age, especially for Chinese male individuals. The longitudinal cohort curve also suggested that the attributable stroke mortality for both genders in both countries was higher in earlier birth cohorts. Those findings all suggested that the attributable stroke mortality was associated with age. Previous studies have proved that age is one major non-modifiable risk of stroke (51). Studies conducted in China and South Korea suggested that stroke mortality is higher in the older population (52, 53). In recent studies, along with the growth of age, stroke mortality is continuously rising in China and South Korea (54, 55).

However, for both genders in South Korea, we also found a mild decline in attributable stroke mortality for age-groups after 85–89 years. A previous study showed that the senior population in South Korea had significant physical changes such as deterioration of oral conditions, decreased chewing function, weakened digestive function, and loss of taste or appetite due to psychological factors (56). All these changes would make it difficult for the elderly (older than 65 years) to eat as much meat as the younger population (56). A previous study also found that compared with the younger population, the senior population had less red and processed meat intake (57), which might be associated with the decline observed in the present study. The reduction of red meat intake might result in the slightly decreased attributable stroke mortality rates in the aged population after 85–89 years.

In both countries, we found that stroke mortality risk for male individuals is higher than that of their female counterparts, and a significant gender difference was observed. Gender differences in stroke mortality have long been observed, while the related mechanism still remained unclear in the current literature (58). However, previous studies may provide some possible explanations. A study conducted in China showed that male individuals usually have higher red meat intake than female individuals (54 vs. 43 grams per day) (59), which may increase the risk of HRMI attributable stroke mortality risk. In addition, female individuals usually had stronger health awareness and more preferred to change their unhealthy lifestyles than male individuals (60), which may make female individuals easier to get knowledge and information about healthy living and change unfavorable habits or lifestyles. Therefore, we propose a hypothesis that once people have controlled the consumption of red meat, especially male individuals (61), the risk of stroke would significantly decrease.

During 1990–2019, ASMRs showed an overall mild fluctuated rising trend for male individuals, while a decreasing trend for female individuals in China. A previous study in China also showed that the stroke mortality trend is volatile, and two peaks in the past three decades appeared in 2005 and 2010 (62), which is similar to the findings of this study. Compared with China, South Korea had lower stroke mortality and lower red meat consumption (63–65), which may result in a decline in ASMRs. The trend declined slowly before 2000, after which the declining trend became rapid. This might be due to guidance in diet about reducing the red meat intake being published in 2002 (66). A previous Korean study found that compared with 1998, beef and poultry consumption significantly decreased, from 39 to 31 grams and from 30 to 27 grams per capita per day per kilocalorie, respectively (67).

The period RR for stroke mortality in China for male individuals decreased from 2004 to 2019, and increased from 1994 to 2004, while for female individuals, the period RR showed deceasing trend from 1994 to 2019. According to the China Statistical Yearbook (68), the change in the period effect is parallel and consistent with the changing trend of red meat consumption from 1990 to 2005 in China. From 2005 to 2019, the consumption of red meat is stable in China (68), while the HRMI attributable stroke mortality keeps dropping for both genders. This may be partly because of the development of socioeconomic status in China, the improvement of residents’ living conditions, and the enrichment of medical services.

The period RR for both genders in South Korea has been declining in the past three decades and fell below 1 in 2004. With the development of the socioeconomic status, the consumption of red meat increased over time. However, in 2002, a diet guideline (66) has been officially published and suggested that the intake of red meat should be limited. The rapid decline of the period RR may be associated with the publishing of the diet guideline, which also suggested that proper dietary guidance may have a positive role in promoting healthy eating.

Nowadays, red meat is leaner and lower in fat content than that 10 years ago (69). This is mainly the result of the changes in animal production and dietary patterns combined with butchery techniques (70). A previous study showed that lean red meat did not elevate markers of oxidative (71) and abdominal obesity (72), and less affect blood pressure (73). Although there is no research evidence on the association between lean red meat and the risk of stroke, we may speculate that fatty red meat may have a more negative effect on stroke than lean red meat. Therefore, the control of fatty red meat intake may be of greater importance.

The cohort effect showed consistent downward trends in both genders in both countries. The cohort effect is dependent on age and period effects; indeed, it is hard to explain them separately in the real world. Recently published APC analyses of stroke mortality attributable to diet (74) or hypertension (75) have suggested the same decline trend in the cohort effect. As mentioned earlier, younger generations (people born in the later birth cohorts) had a lower risk and mortality of stroke. Considering the dependency between age and cohort effects, this result is consistent. The living, dietary, and medical conditions of the younger generations are better than those of the older generations, and in addition, the younger generations are more educated and have higher health knowledge and awareness; these may all result in the decline in the attributable stroke mortality risk (76).

Through literature review, we found this study is the first research focusing on the temporal trends of stroke mortality attributable to HRMI and providing a comprehensive quantified comparison between China and South Korea. However, there are also some limitations to this work. Because the GBD data were summarized statistics, substantial bias may not be totally avoided. Future cohort-based studies may be of great value to verify our findings. Then, due to the complexity of the diet in China and South Korea, the estimation of the red meat intake amount may also be influenced by potential bias. In addition, the results obtained using the data from China and South Korea may not be able to extend to other countries in the same region.

## Conclusion

In summary, the mortality of stroke due to high consumption of red meat is more serious in China than in Korea. In both the countries, male individuals had a higher risk of stroke than their female counterparts. Male individuals, especially middle-aged to old male individuals in China, are at higher risk of HRMI attributable stroke risk. Our findings suggested that controlling the intake of red meat could be a cost-effective strategy to reduce stroke risk and the corresponding disease burden.

## Data availability statement

Publicly available datasets were analyzed in this study. This data can be found here: http://ghdx.healthdata.org/.

## Author contributions

JM and FC: conception and writing – critical revision. FC and WH: data acquisition. WH and SC: software. FC, JM, and WH: formal analysis and writing – original manuscript. WH, SC, AS, and YZ: investigation. All authors contributed to the article and approved the submitted version.

## Footnotes

1
http://ghdx.healthdata.org/

2
https://analysistools.nci.nih.gov/apc/
